# La pneumonie tuberculeuse: une nouvelle série de 27 cas

**DOI:** 10.11604/pamj.2014.19.122.5178

**Published:** 2014-10-03

**Authors:** Ouiam Bakouh, Sarra Aniked, Jamaleddine Bourkadi

**Affiliations:** 1 Service de Pneumo-phtisiologie, Hôpital Moulay Youssef, CHU Rabat, Rabat, Maroc

**Keywords:** Pneumonie tuberculeuse, tuberculose aigue, VIH, Pneumonia tuberculosis, acute tuberculosis, HIV

## Abstract

Dans le but d’étudier les aspects cliniques, radiologiques et évolutifs de la pneumonie tuberculeuse (PT) au Maroc, pays à forte prévalence de tuberculose, une étude rétrospective s’étalant de Janvier au Septembre 2013 a été menée au service de phtisiologie femme de l'hôpital Moulay Youssef de Rabat. 27 cas de PT ont été diagnostiqués, dont 2 VIH séropositives. La fièvre et l'altération de l’état général étaient rapportées chez toutes les patientes, précédant les signes respiratoires comme La toux et la dyspnée. L'hémoptysie est rapportée chez 7 cas. Diagnostiquées souvent tardivement, du fait de la non spécificité de ses signes, 14 pneumonies sur 27 étaient excavées. Avec prédominance des lésions au lobe supérieur droit. Le traitement antituberculeux était efficace dans la majorité des cas. On a déploré 2 décès. La décision de mise en route du traitement antituberculeux même en l'absence de certitude bactériologique doit être prise dans un délai raisonnable de 15 jours vue la gravité du tableau et les séquelles persistantes.

## Introduction

La pneumonie tuberculeuse (PT) ou caséeuse est une forme aigue et rare de la tuberculose pulmonaire. Le diagnostic différentiel entre PT et pneumonie communautaire est difficile souvent source de retard diagnostic. Son pronostic est parfois réservé dépendant de la rapidité de la prise en charge. Compte tenu de la gravité de la maladie et de ses séquelles, en l'absence de certitude bactériologique, l'association de terrain débilité, altération de l’état général avec amaigrissement, fièvre, absence d'amélioration après antibiothérapie probabiliste devrait faire évoquer le diagnostic et mettre en route le traitement antituberculeux.

## Méthodes

Il s'agit d'une étude rétrospective effectuée en service de phtisiologie femme de l'hôpital Moulay Youssef, s’étalant de Janvier à Septembre 2013, ayant colligée 27 cas.

## Résultats

**Figure 1:**
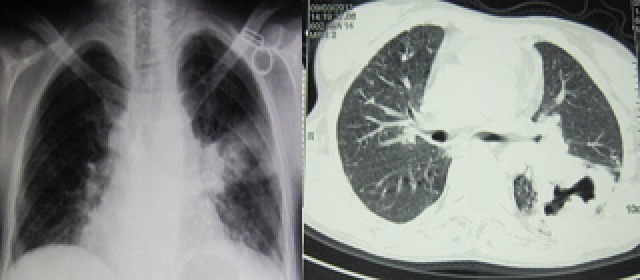
Radiographie et scanner thoraciques d'une patiente de 40 ans montrant une PT du lobe inferieur gauche

**Figure 2:**
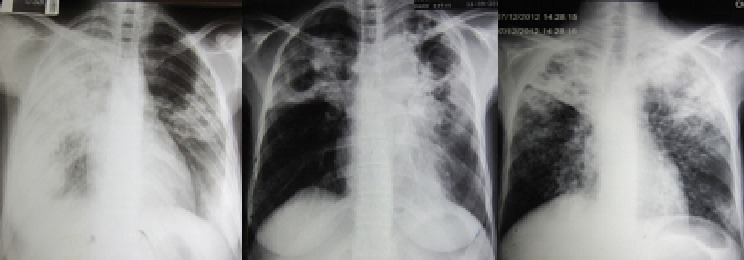
Radiographies thoraciques montrant des PT bilatérales

**Figure 3:**
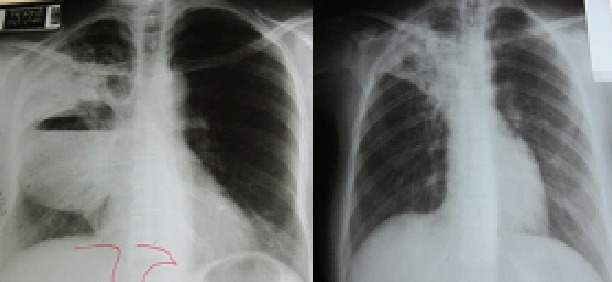
Radiographies thoraciques montrant des PT excavées du poumon droit

La moyenne d’âge est de 39 ans. Le tabagisme est retrouvé chez 6 cas, le diabète chez 5 cas, 1 cas d'insuffisance rénale chronique en stade d'hémodialyse et 2 cas de VIH+ et 1 cas d'une patiente sous corticothérapie orale au long court pour un lupus érythémateux disséminé. La notion de contage tuberculeux est retrouvé dans 5 cas Le délai moyen du diagnostic est de 6 semaines, avec installation de signes généraux en premier à type de fièvre, altération de l’état général chez tous les cas, puis, l'apparition de façon aigüe de signes respiratoires à type de toux, dyspnée et douleur thoracique chez toutes nos patientes, hémoptysie chez 7 cas. La radiographie thoracique a objectivé une opacité alvéolaire systématisée située: à droite chez 23 cas (85%) avec prédominance de l'atteinte du lobe supérieur droit, et à gauche chez 3 cas, (Figure 1) bilatérale chez 3 cas (Figure 2). Les excavations sont présentes chez 14 cas (51,8%) (Figure 3). L'antibiothérapie probabiliste a été démarrée avant l'hospitalisation chez 11 cas. Le diagnostic positif est bactériologique par la mise en évidence du Mycobacterium tuberculosis à l'examen direct des crachats dans tous les cas. Toutes les patientes sont traitées par les anti bacillaires a base de 2 mois de rifampicine (10 mg /kg /jour), l'isoniazide (5 mg /kg /jour), Éthambutol (15-20 mg /kg /jour) et pyrazinamide (25-30 mg /kg /jour) suivi d'une phase de quatre mois de rifampicine et Isoniazide avec une évolution favorable dans 21 cas, retard de conversion de l'examen direct des crachats jusqu'au 3eme mois du traitement chez 4 cas ayant toutes des formes étendues, on a déploré 2 cas de décès.

## Discussion

La pneumonie tuberculeuse (PT) est une forme rare de la tuberculose pulmonaire, mais n'est pas exceptionnelle en zone de forte prévalence de tuberculose. Il s'agit d'une infection tuberculeuse massive d'un lobe ou d'un poumon évoluant spontanément vers la fonte caséeuse. Son incidence varie selon les séries. Dans une série d'Ouedraogo, parmi les 106 patients atteints de tuberculose pulmonaire 26 pneumonies caséeuses ont été rapportées (24,5%) [1]. Nyamande et al. rapportent 39,6% de cas de pneumonie due au mycobacterium tuberculosis parmi les 222 patients hospitalisés pour pneumonie aigüe communautaire (PAC) [2]. Une incidence plus faible, estimée à 9% est rapportée dans les séries de Scott [3] et Kamanfu [4]. Elle affecte surtout le sujet jeune, dans la série d'Ouedraogo 61,5% des patients avaient moins de 45 ans [1]. La moyenne d’âge est de 33 ans dans la série de Naman [2] concordant avec notre série ou la moyenne d’âge est de 39 ans. Des terrains particuliers de prédisposition à développer une pneumonie tuberculeuse avec augmentation du risque de réactivation d'une tuberculose latente tel que le diabète, l'insuffisance rénale chronique, une gastrectomie, des conditions d'immunodépression (VIH+, corticothérapie au long court ou chimiothérapie ou une biothérapie) sont souvent retrouvés [5]. Dans notre série, ces facteurs prédisposants sont retrouvés chez 9 cas (33%).

Le mycobactrium tuberculosis est l'agent infectieux le plus retrouvé dans la pneumonie aigue du patient VIH +. Dans la série de Nyamande, 40% des patients étaient VIH+ [2]. Grant et al. Rapportent que 61% des patients HIV+ hospitalisés en service des maladies respiratoires de l'hôpital d'Abidjan avaient une pneumonie tuberculeuse [6]. Dans notre série, nous rapportons 2 cas de VIH +. La notion de contage tuberculeux doit être recherchée à l'interrogatoire permettant d'orienter le diagnostic.

Le diagnostic précoce de la pneumonie tuberculeuse n'est pas toujours aisé, partageant très souvent le même masque radio-clinique que les pneumopathies à germes banals, ce qui rend le diagnostic souvent tardif. Le délai diagnostic dans la série de Ouedraogo, a varié de 1 à 32 semaines avec une moyenne de 4 semaines [1], s'approchant du délai de diagnostic moyen dans notre série qui est de 6 semaines Dans la série de Pinto il est seulement de 10 jours, mais avec un tableau clinique plus bruyant [7].

Les signes cliniques ne sont pas spécifiques, tels que la fièvre, l'asthénie, l'amaigrissement et l'anorexie, rapportés chez la majorité des patients. Les signes fonctionnels respiratoires sont dominés par la toux, présente chez tous les patients, la douleur thoracique et la dyspnée chez une grande partie des patients. L'hémoptysie est rapportée dans 11,5% des cas d'Ouedraogo, sans différence selon le VIH [1]. Et dans 25% (7 cas) de notre série. L'incidence de la détresse respiratoire aigue sur pneumonie tuberculeuse est rapportée dans 1,5% des cas de patients hospitalisés pour tuberculose pulmonaire [8]. Sur le plan radiologique, on constate une opacité systématisée, pouvant être excavée ou non. L'atteinte du lobe supérieur droit est la plus fréquente, retrouvée dans 67,8% dans la série d'Ouedraogo et dans 83,2% dans la série de Tidjani [9]. Dans la série d'Ouedraogo [1], il n'a pas été signalé de différence dans les aspects radiologiques entre les patients VIH + et ceux VIH-. Certaines études notent las rareté des excavations chez les sujets immunodéprimés [10].

La recherche de BAAR à l'examen direct des crachats est l'examen de première intention chez l'adulte devant toute suspicion de tuberculose pulmonaire. Son rendement n'est pas constant surtout au cours de la pneumonie caséeuse. L'originalité de la pneumonie caséeuse réside dans la difficulté de mise en évidence des BAAR, incitant à la répétition de l'examen. Si l'examen direct n'est pas contributif, d'autres examens comme le tubage gastrique ou le lavage broncho alvéolaire et la mise en culture de prélèvements sont souvent indispensables pour mettre en évidence le bacille tuberculeux [1]. Dans notre série, le diagnostic positif est bactériologique par la mise en évidence du Mycobacterium tuberculosis à l'examen direct des crachats dans tous les cas.

Dans certains cas de pneumonie tuberculeuse, le patient est mis d'abord sous antibiothérapie probabiliste à base de fluoroquinolones, ce qui en résulte en une amélioration transitoire du tableau clinique, induisant un retard dans le diagnostic et dans l'institution d'un traitement antituberculeux. Ces retards sont connus pour entraîner une augmentation de la morbidité et de la mortalité, et du risque de transmission [11]. Aussi si la première antibiothérapie doit être large, la décision de mise en route du traitement antituberculeux même en l'absence de certitude bactériologique doit être prise dans un délai raisonnable de 15 jours [1]. Le traitement de la tuberculose pulmonaire ne change pas selon la sévérité de la présentation initiale. La présence d'insuffisance respiratoire ne justifie pas une modification du traitement de la tuberculose qui comprend une phase d'attaque, composé de 2 mois de rifampicine (10 mg /kg /jour), l'isoniazide (5 mg /kg /jour), Éthambutol (15-20 mg /kg /jour) et pyrazinamide (25-30 mg /kg /jour) suivi d'une phase de quatre mois de rifampicine et Isoniazide. Les effets bénéfiques de l'addition de corticostéroïdes en cas d'insuffisance respiratoire ne sont pas prouvés ni recommandés [12].

Le pronostic de la pneumonie caséeuse, indépendamment du terrain est fonction de la précocité du diagnostic et du traitement. Dans la série d ‘Ouedraogo, une bonne évolution a été constatée chez la majorité des patients, avec un taux de conversion après 2 mois de 77,7% chez les VIH+ et de 82,3% chez les VIH -. 2 cas de décès ont été rapportés [1]. Dans notre série, le taux de conversion après 2 mois est de 77,7% (21 cas) avec retard de conversion chez 4 cas qu'après 3 mois du traitement anti bacillaire, on a déploré également 2 décès par sepsis chez une patiente VIH + et une autre suivie pour lupus érythémateux disséminé sous corticothérapie au long court. Dans la série de Scott, la mortalité de la pneumonie tuberculeuse était de 10% [3]. Dans une série camerounaise comparant la pneumonie tuberculeuse entre le groupe de malades VIH+ et le groupe des VIH-, il n'a pas été constaté de différence dans la mortalité entre les 2 groupes [13].

## Conclusion

Pour évaluer la probabilité de la tuberculose chez un patient atteint d'une pneumonie communautaires, certains paramètres cliniques et biologiques doivent être pris en considération: la chronicité des symptômes, la présence des épanchements pleuraux, réponse insatisfaisante à une antibiothérapie empirique qui couvrent les agents pathogènes les plus communs, l'amélioration transitoire après utilisation de fluoroquinolones suivie d'une détérioration après l'arrêt du traitement, des antécédents de tuberculose ou une notion de contage, un taux normal de leucocytes devant alerter le médecin de la possibilité de la tuberculose.
